# The spider fauna from Uruguay River islands: understanding its role in a biological corridor

**DOI:** 10.3897/BDJ.6.e27319

**Published:** 2018-08-28

**Authors:** Álvaro Laborda, Laura Montes de Oca, Fernando Pérez-Miles, Gonzalo Useta, Miguel Simó

**Affiliations:** 1 Sección Entomología, Facultad de Ciencias, Universidad de la República. Iguá 4225. CP 11400, Montevideo, Uruguay Sección Entomología, Facultad de Ciencias, Universidad de la República. Iguá 4225. CP 11400 Montevideo Uruguay; 2 Instituto de Investigaciones Biológicas Clemente Estable, Laboratorio de Etología, Ecología y Evolución. Av. Italia 3318. CP 11200, Montevideo, Uruguay Instituto de Investigaciones Biológicas Clemente Estable, Laboratorio de Etología, Ecología y Evolución. Av. Italia 3318. CP 11200 Montevideo Uruguay; 3 Laboratorio Tecnológico del Uruguay. Av. Italia 6201. CP 11500, Montevideo, Uruguay Laboratorio Tecnológico del Uruguay. Av. Italia 6201. CP 11500 Montevideo Uruguay

**Keywords:** Biodiversity, Biogeography, Araneae, regional connections

## Abstract

Biological corridors are connections which link habitats in a regional scale, allowing the gene flow between populations. The Uruguay River comprises riverside and insular riparian forests along subtropical to temperate zones passing through different biogeographic provinces. The aim of this study was to characterise the spider fauna from the Uruguay River islands highlighting their connection role for the spider community of riparian forest. Spiders were studied from surveys in a fluvial island of the southern course of the river with nine campaigns being carried out from September 2007 to September 2009. Three complementary collecting methods were used: G-Vac, night hand collecting and pitfall traps. A total of 58 samples were taken in each campaign. A total of 33 families, 145 species/morphospecies and 8 guilds were registered. Theridiidae and space web weavers showed the highest abundance and species richness. Web weavers were predominant in the spider community evidencing the importance of the forest vegetation heterogeneity in spider diversity. Fifteen species have been recorded for the first time for Uruguay. Additional data of previous surveys in the northern islands of the river were analysed and compared. Several species confirm the role of the Uruguay River as a biological corridor from the upper to lower course of the river. The riparian forests from the islands constitute a southernmost intromission of Paranaense biota between Chaco and Pampa regions along the river. The results obtained are an important input for the conservation of these areas. Knowing the biodiversity, as well as its dynamics and the flow of biota that exists in these environments, would allow planning the management from a regional point of view.

## Introduction

Connections between natural environments allow gene flow through migrants, essential to maintain viable populations (Cushman et al. 2006, Christie and Knowles 2015, Fahrig 2003, Sharma et al. 2013) and to provide suitable areas along their geographic distribution range (Heller and Zavaleta 2009). Biological corridors are continuous patches of vegetation which allow the movement of individuals amongst habitats, mitigating the effect of fragmentation and preventing the isolation of populations (Merriam 1984). For these reasons, the maintenance of biological corridors is a valuable conservation strategy to protect biological diversity (Saunders and Hobbs 1991). Knowledge of connections between environments and the biota exchange is essential for planning the accurate management of natural areas and avoiding habitat fragmentation processes (Evans et al. 2013, Kormann et al. 2016, MacClintock et al. 1977). Studies on connections and interactions between the biota are considered as a crossroad where biogeographical provinces converge (Morrone 2017), creating a mosaic of environments in a relatively small territory (Grela 2004, Simó et al. 2014, Simó et al. 2015). One of these convergences occurs especially in the riparian forests of the Uruguay River, where the dendroflora presents components of the biogeographic provinces of Chaco and Paranaense Forest (Grela 2004).

The Uruguay River is the most important fluvial course in the Río de la Plata basin, after the Paraná River, with approximately 1770 km of length. It originates in southern Brazil and empties into the Río de la Plata. Along its course, dense riverside forests and numerous islands formed by alluvial deposits exist (DINAMA 2014). The Uruguay River basin comprises territories from Argentina, Brazil and Uruguay covering a total area of about 339,000 km^2^ serving as the international boundary between these countries (CARU 2014). Most of this area has been modified in the last centuries due to different human activities, such as urbanisation and agricultural-livestock production (DINAMA 2014). This anthropic intervention has caused a drastic reduction and fragmentation of natural environments such as the riparian forest of the Uruguay River and its tributaries (Brussa and Grela 2007). These threats, together with their high species richness and the valuable ecosystem services provided, are the reasons for considering these forests as priority areas for conservation (Brazeiro et al. 2015).

However, not all areas with riparian forest have suffered the same degree of alteration. The human impact on Uruguay River islands is mitigated by its difficult access and many of them are still well conserved (DINAMA 2014). As these islands are constituted by contributions from the river sediments, they are therefore dynamic and their physiognomy changes by erosion processes and sediment accumulation (DINAMA 2014). These processes can be extreme in some cases, such as the periodic flooding of the river, which sometimes can completely submerge the islands (DINAMA 2014). The river is the architect of the islands, which, not only determines its physical form, but also its biotic composition. The islands receive a constant contribution of seeds and plant parts from the upper course where animals are transported. These elements of propagation across the river are the way for many species to colonise and settle down in new environments (Brussa and Grela 2007, DINAMA 2014, Gutiérrez et al. 2015, Laborda 2012).

Despite this, the biodiversity on the Uruguay River islands has not been thoroughly studied. Some available data belong to riverside forest areas with interest for conservation and tourism and refer mainly to dendroflora and vertebrates (DINAMA 2014, Mello et al. 2008). These studies indicate that these forests represent relictual environments which preserve complex communities and ecological processes along the river. Therefore, the study of their biota is crucial for the environmental management of these areas at local and regional level.

Undoubtedly, the study of megadiverse groups are of major interest in these areas because they have high species richness and play important roles in ecosystems. The Order Araneae, with more than 47000 species described (WSC 2018), is a megadiverse group of predators (Coddington and Levi 1991, Wheeler et al. 2017), abundant in terrestrial ecosystems where they regulate herbivore populations and occupy a strategic place in trophic networks (Ferris et al. 2000, Lawrence and Wise 2000). This strategic position makes them regulators of the dynamics of energy and nutrient flow in terrestrial environments (Van Hook 1971, Zeische and Roth 2008).

The only previous spider survey in islands and riverside forests from the Uruguay River has been made within the influence area of Salto Grande dam (northern Uruguay) before inundation (Pérez-Miles 1988). This contribution provided a spider checklist with few records from islands. However, it represents a great testimonial value because the environments surveyed are currently totally or partially submerged.

The aim of this study is provide data about the composition and structure of the spider community from the insular riparian forest of the Uruguay River in a biogeographic framework. The subtropical characteristics of the islands' vegetation and previous studies evidence that the southern course of the river constitutes the southernmost distribution limit for some spider species (Laborda et al. 2012, Machado et al. 2013). Consequently, we hypothesise that the insular spider fauna will present components from upper subtropical zones, as an extension of the Paranaense Forest influence between Chaco and Pampa provinces, as was proposed for the dendroflora (Grela 2004). We also propose that the Uruguay River could function as a biological corridor between Northern and Southern riparian environments.

## Material and methods

**Figure 1.**
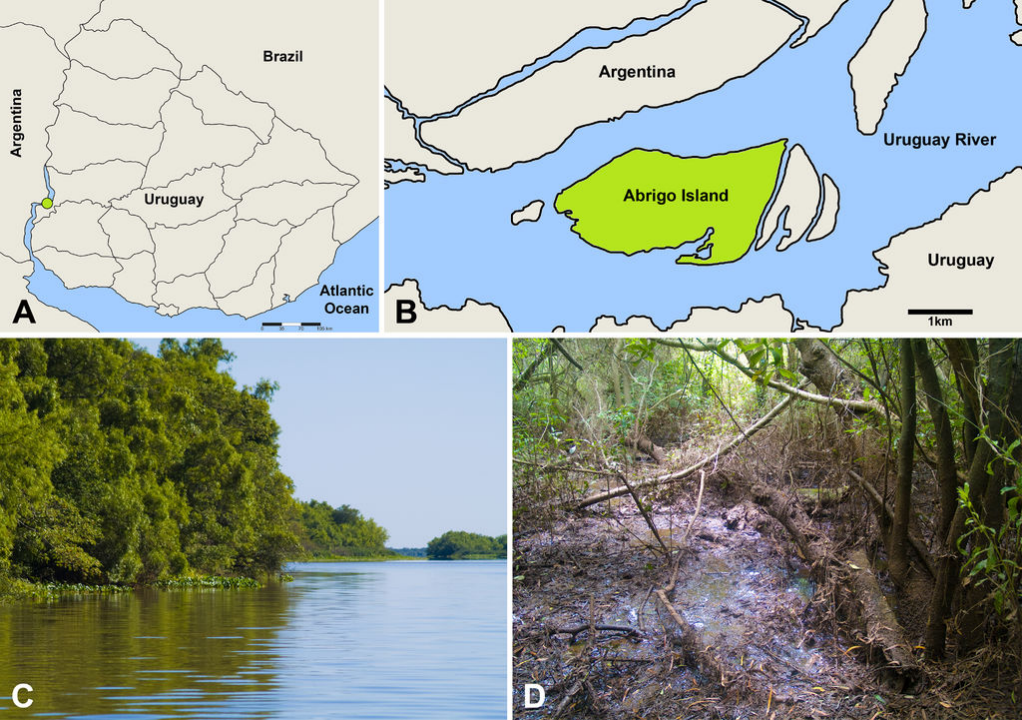
Study area. **A**: Location of the study area in the Uruguay River; **B**: Abrigo Island; **C**: Riparian forest of Abrigo Island, view from the river; **D**: View from the inside of the riparian forest in Abrigo Island.

The study area was located in Abrigo island, upstream of the General San Martin International Bridge (33°5'13.75"S; 58°10'38.55"W) (Fig. 1A and B), 10 km northwest of Fray Bentos city. The island is about 1.6 km² and is approximately 700 m from the Uruguayan coast. Like other islands of the Uruguay River, its origin is a consequence of the discharge and sediments accumulation transported by the river (DINAMA 2014) and the vegetation matrix is a dense native forest, with subtropical characteristics (Fig. 1C and D). The main representative plant species are: *Guadua chacoensis* (Rojas) Londoño and P.M. Peterson (Poaceae), *Hexachlamys edulis* (O.Berg) Kausel and D.Legrand (Myrtaceae), *Inga vera* Mart., *Lonchocarpus nitidus* (Vogel) Benth., *Albizia inundata* (Mart.) Barneby and J.W.Grimes (Fabaceae), *Peltophorum dubium* (Spreng.) Taub. (Leguminosae), *Handroanthus heptaphyllus* (Vell.) Mattos (Bignoniaceae), amongst others (Brussa and Grela 2007).

The surveys were carried out every three months from September 2007 to September 2009, making a total of nine campaigns. Three complementary collecting methods were used, in order to sample the different strata in the environment: G-Vac, night hand collecting and pitfall traps. A total of 58 samples were taken in each campaign, including: 10 pitfall traps, 8 hand collections, 40 G-Vac aspirations (one-minute duration each one) 20 during the day and 20 during the night (10 from soil and 10 from foliage in each one). A total of 522 samples were taken during the entire survey period.

The pitfall traps consisted of plastic containers of 22 cm in diameter and 12 cm height, buried and covered with a plastic roof supported by three metallic rods 10 cm above the soil. The traps were placed 10 m apart from each other along a transect line of 100 m parallel to the coast. A mixture of 7% formaldehyde and detergent was used as fixative solution. All traps remained active during 30 days. Nocturnal hand collecting involved four collectors and was performed using head lamps, during 30 minutes, following the ‘looking up and looking down’ method (Coddington et al. 1996). To know the spider composition, the specimens were identified at the family level using a key (Grismado et al. 2014) and after, to a species level using taxonomic literature (WSC 2018). If it were not possible to determine the species, we used morphospecies, taxonomic units widely used in diversity works on arthropods (Derraik et al. 2002, Oliver and Beattie 1996).

A photographic database was elaborated for the species/morphospecies recognition. Dorsal and ventral habitus photographs, as well as female and male genitalia, were taken using stereoscopic microscopes. The collected specimens were preserved in 70% alcohol and vouchers were deposited in the arachnological collection of the Facultad de Ciencias, Universidad de la República (FCE). Furthermore, specimens deposited in this collection from previous surveys in islands from Uruguay River were considered for comparison with the results here obtained. Guild classification was based on Cardoso et al. (2011), considering it as the most recent proposal and it comprises the worldwide spider families.

The sampling efficiency was estimated with EstimateS 9.1.0 (Colwell 2006), using the non-parametric richness estimator Chao1 (Toti et al. 2000), this estimator being selected because the normality assumption was not satisfied. For the diversity settings, we applied 500 randomisations of sample order.

The species obtained were classified in decreasing order of abundance and these data were graphed and compared with four mathematical models of abundance distribution (geometric, log series, log-normal and broken stick) in order to determine the best fit of the data collected. The fit was determined using Chi-square. Significance level of 0.05 was used (Magurran 1988). To compare the capture methods and guilds, Chi-square tests of "goodness of fit" were made. Using a null hypothesis, a uniform distribution was assumed. Significance level of 0.05 was used. The statistical analysis and the graph were made using Past (Hammer et al. 2001)

The map was elaborated using SimpleMappr (Shorthouse 2010).

## Results

A total of 7605 spiders were collected, distributed in 33 families and 145 species/morphospecies (Table 1).

From the total number of specimens collected, 79% were juveniles (n=5985), 12% adult females (n=909) and 9% adult males (n=711).

Most of the collected specimens (80%) belong to seven families, Theridiidae (n=1777; 23%), Araneidae (n=1400; 18%), Anyphaenidae (n=703; 9%), Lycosidae (n=687; 9%), Salticidae (n=584; 8%), Thomisidae (n=511; 7%), Linyphiidae (n=475; 6%), the remaining 26 families representing 20% (n=1468) of the total abundance found.

Four families comprise more than half of the registered species, Theridiidae (S=35; 24%), Linyphiidae (S=17; 12%), Araneidae and Salticidae (S=16; 11%) and Thomisidae (S=10; 7%), the rest of the families having less than 10 species.

The most abundant species were: Theridiidae sp1, *Aysha* sp.1 (Anyphaenidae), *Eustala photographica* (Araneidae), *Lobizon humilis* (Lycosidae) and Hahniidae sp.1. Forty-three species were singletons, comprising 30% of the sampled species.

Chao 1 estimator indicated 187.97 species for the studied site, which means that the species recorded represent 77.14% of the estimated species richness for this environment.

**Figure 2.**
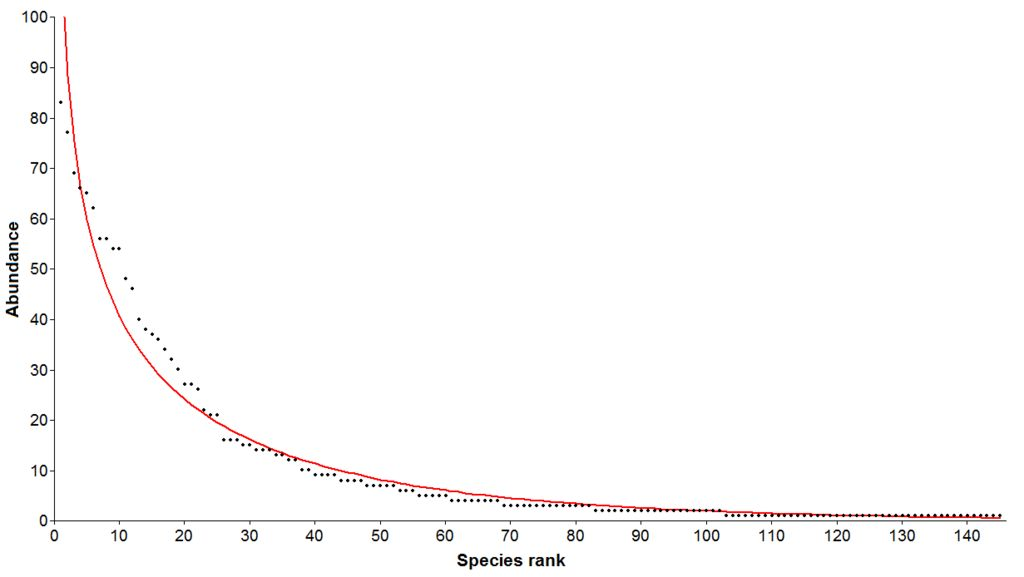
Log series model of the abundance distribution of spiders in Abrigo Island (χ2=51.38; p=0.999).

The best fit for the abundance distribution of the spider community was the log series model (χ2=51.38; p=0.999) (Fig. 2).

Fifteen of the registered species represent new records for Uruguay: *Otoniela quadrivittata* (Anyphaenidae); *Dubiaranea difficilis*, *Scolecura parilis*, *Sphecozone venialis* (Linyphiidae); *Agalenocosa pirity*, *Lobizon corondaensis* (Lycosidae); *Mimetus melanoleucus* (Mimetidae); *Xiombarg plaumanni* (Oonopidae); *Architis capricorna* (Pisauridae); *Cotinusa trifasciata*, *Synemosyna aurantiaca* (Salticidae); *Leucauge volupis* (Tetragnathidae); *Cryptachaea altiventer*, *Cryptachaea bellula* (Theridiidae); *Uloborus elongatus* (Uloboridae).

The greatest abundance was obtained with the G-Vac method (n=5034; 66%) (p=0.0001) followed by hand collecting (n=1563; 21%) and pitfall traps (n=1008; 13%), the same pattern being observed when only adults (p=0.0001), G-Vac (n=690; 43%), hand collecting (n=486; 30%) and pitfall traps (n=444; 27%) were considered.

Representatives from eight guilds were found: ground hunters (GH), ambush hunters (AH), sensing web weavers (SEW), space web weavers (SPW), orb web weavers (OW), sheet web weavers (SHW), specialists (S) and other hunters (OH). The weavers spiders guilds showed a significantly higher abundance (p=0.0001), but no significant differences in species richness were found (p=0.1) (Table 2).

The GH and the SHW were more abundant in the soil samples of G-Vac and in the pitfall traps (p=0.0001), the most part of the OW being obtained by hand collecting (p=0.0001) and the OH and SPW were more abundant in the G-Vac samples (p=0.0001). No significant differences per method were observed in the others guilds.

## Discussion

Species richness and abundance (7605 individuals, 145 species and 33 families) reached high values compared with surveys carried out in the country, such as in hilly environments (Costa et al. 1991, Pérez-Miles et al. 1999), in sandy coasts (Costa et al. 2006) and natural grasslands (Laborda 2012). Despite the differences in collection effort and methods used on these studies, the results obtained in this work suggest that spider fauna in the Abrigo island from Uruguay River is highly diverse.

Chao 1 estimator indicated that 77% of the spider species have been registered in this study. According to Cardoso (2009), surveys range between 70–80%, indicating we have achieved a comprehensive inventory. About 23% of the species remains to be known, which means that additional studies are needed to advance the knowledge of the species that inhabit a complex and changing environment like the riparian forests in the Uruguay River islands. From all the collected individuals, 21.3% were adults, in agreement with Duffey (1962) and Breymeyer (1966) who report that adults do not exceed 48% of the natural populations in Araneomorphae and are similar to values obtained in others surveys conducted in Uruguay using diverse collecting methods (Laborda 2012).

In riparian habitats, the disturbance promoted by flooding produces extinction and posterior species recolonisation through floating vegetation (Paetzold et al. 2008, Schiesari et al. 2003). According with our results, the abundance distribution of the spiders' community conformed to a log series model (Fischer et al. 1943), where the species arrive at an unsaturated habitat at irregular intervals of time (Magurran 1988). This is consistent with the dynamics of the Uruguay River islands. The floods of the river reduce the islands' surface area or can completely submerge them causing a major disturbance in the system. After the floods, when the water level drops, animals and plants are transported in floating vegetation from the upper course of the river, thus, recolonising the islands.

The infra-order Mygalomorphae was not recorded in the present study. This can be explained by the recent sedimentary origin of the island Abrigo (DINAMA 2014) and, considering the limited dispersion capacity of most species of tarantulas (Ferretti et al. 2010, Satler et al. 2013), it is possible that these recent and changing environments are difficult to colonise by this group. At the most southerly point, on Martín García Island, there are records of Mygalomorphae (Ferretti et al. 2010); however, it is a much older island with a different origin, related to continental geological formations. Another explanation is related to the incidence of the water level increase. Periodical floods of the river do not affect Martin Garcia Island because its surface rises around 27 m a.s.l., but flooding partially or totally covers the surface of the Abrigo Island which is only 3-5 m a.s.l. (DINAMA 2014).

The families Anyphaenidae, Araneidae, Linyphiidae, Lycosidae, Salticidae, Theridiidae and Thomisidae reached high values of species richness and abundance. These families constitute extremely diverse and widely spread groups in the world (WSC 2018). Furthermore, this result agrees with other studies performed in the country (Costa et al. 2006, Laborda 2012). An unidentified small Theridiidae was the most abundant species, collected in pitfalls and ground samples of G-Vac, thus indicating a ground-level habitat. The fourth and fifth species in abundance were also present at ground-level: *Lobizon humilis* and Hahniidae sp.1, which shows the importance of the low strata in the abundance of spiders in these environments. The second and third species in abundance, *Aysha* sp.1 and *Eustala photographica*, are a foliage hunter and an orb weaver, respectively, that use the abundant and complex vegetation for hunting and to construct their webs.

The fifteen new species records for Uruguay indicate a great diversity of these environments and the knowledge gaps that exist in the distribution of spider species. *Agalenocosa pirity* and *Lobizon corondaensis* are small-sized wolf spiders, associated with wetlands and flood forest environments (Piacentini 2014, Piacentini and Grismado 2009). *Architis capricorna* is also an inhabitant of the forest floor in semi-aquatic habits, always associated with watercourses (Santos 2007, Santos and Nogueira 2008). Very little is known about the natural history of *Cotinusa trifasciata*. In this study, this species was collected with G-Vac in the tree foliage in spring and summer. *Synemosyna aurantiaca* is a mimetic species with an ants' appearance and behaviour of the genus *Pseudomynnex*, which build their nests in the vegetation (Galiano 1966). *Cryptachaea altiventer* and *Cryptachaea bellula* are space web weaver’s spiders that inhabit the foliage of riparian forest trees; in particular, these two species have been reported in Argentina for similar environments, close to the study area/site (Grismado et al. 2011). *Scolecura parilis* and *Sphecozone venialis* are species that construct small sheet webs near the ground and were collected with pitfall traps and G-Vac on soil. These species have been recorded for tropical and subtropical environments in southern Brazil and northern Argentina (Grismado et al. 2011, Miller 2007). Another new record from the Linyphiidae family is *Dubiaranea difficilis*, which builds sheet webs amongst herbaceous vegetation a few centimetres above the soil, this species having been registered for mountain forests and rainforests in Argentina (Rubio et al. 2010). *Leucauge volupis* builds horizontal orb webs in the lower strata of the forest and has been reported from southern Brazil (Buckup et al. 2010, Ott et al. 2007). Nothing is known about *Mimetus melanoleucus* natural history also recorded from southern Brazil and northern Argentina (Grismado et al. 2011, Mello-Leitão 1929). As the family is generally characterised for preying exclusively on other spiders, especially on weaver spiders (Foelix 2011), it is therefore expected to find these mimetid spiders in this type of wooded environment, due to the great abundance of potential preys. *Otoniela quadrivittata* was collected in the foliage along with other species of Anyphaenidae, its distribution being very wide, from Venezuela to Argentina (Brescovit 1997). *Uloborus elongatus*, a cribellate orb weaver, was reported for Iguazu Falls in the province of Misiones, a tropical environment of northern Argentina (Opell 1982). Within the Oonopidae, a new record is *Xiombarg plaumanni*, known from southern Brazil and northern Argentina (Misiones) (Grismado and Izquierdo 2014). There is no previous data about the natural history of this species; in our study, it was found in the foliage, in low branches of trees and shrubs and was collected with G-Vac in spring and summer.

The four weaver spider guilds registered (SEW, SPW, OW and SHW) represent the 59% of the individuals collected. The structural complexity of the environment, a mixed forest with several strata, provides numerous physical spaces in which different species construct their webs (Jiménez-Valverde and Lobo 2007, Rubio and Moreno 2010, Scheidler 1990). Space web weavers, mainly represented by Theridiidae, occupy all strata due to their great diversity of forms and habits (Agnarsson 2004). The sensing web weavers, represented only by *Ariadna mollis*, was found in tube webs inside hollow dry branches that are still attached to the trees.

Other guilds registered were the GH and OH, represented by species which do not construct webs to hunt. They are also spatially separated, GH occupying the lower stratum and OH the high strata of vegetation. GH included mostly small species of *Agalenocosa* and *Lobizon* (Lycosidae). These species use the low stratum of the forest and have been reported inhabiting semi-aquatic vegetation in wetland environments and hygrophilous forests (Piacentini and Grismado 2009, Piacentini 2014). This fact agrees with the study area environment, where is regularly flooded. In OH, the majority of families were Anyphaenidae and Salticidae, most of its species are active and fast hunters, inhabit preferably the arboreal foliage and are abundant and diverse in most ecosystems (Jackson and Pollard 1996, Ramírez 2003).

This differential use of the strata is consistent with the significant differences observed in the abundances of the guilds obtained by comparing the collecting methods. It alsoshows the importance of using different sampling techniques to study the spider community, because each method allows us to know a different portion of the community (Coddington et al. 1990).

Specimens of uncommon families such as Dictynidae, Oonopidae, Deinopidae and Senoculidae were registered for the country. Dictynidae was represented in this study by an undetermined species of *Dictyna*. This family has been little studied in the region (WSC 2018) and, in Uruguay, there is only an old record for *Dictyna similis* (Keyserling, 1878). Oonopidae was represented by numerous specimens of several species, including two recently described: *Neotrops lorenae* and *Neotrops sciosciae* (Grismado and Ramírez 2013) and a new record for the country, *Xiombarg plaumanni*. Since this family is being reviewed worldwide, it is extremely important to have representatives in the arachnological collections (The Goblin Spider PBI 2016).

Deinopidae was cited for the country from specimens of *Deinopis amica* collected in the present study (Laborda et al. 2012). Previous records of this species (Schiapelli and Gerschman 1957) link it to the subtropical forests of the northern basin of the Uruguay River. Recently, this species was found at riverside forests in northern Uruguay (manuscript in prep.), which would indicate the existence of a continuous distribution of this species along the Uruguay River. The same scenario is observed in Senoculidae, a family registered for the first time for Uruguay in this study and represented only by juveniles. These two families seem to be closely linked to forests with subtropical characteristics. The same distribution pattern of *D. amica*, associated with the riparian forests of Uruguay River course, is observed for other recorded species such as *Uloborus elongatus, Neotrops sciosciae* and *Mesabolivar uruguayensis*. These findings reinforce the connection role of the river in a biogeographic crossroad (Simó et al. 2014).

The species *Ancylometes concolor* (Perty, 1833) was registered for the study site by a collection record (1♂, FCE Ar-4600). Its presence is additional evidence in favour of the biological corridor hypothesis. The known distribution of the species is: Paraguay, northern Argentina and southern Brazil (Höfer and Brescovit 2000). In Uruguay, it was recorded for northern localities in the Uruguay River, such as Isla Zapallo (30°29'18.68"S; 57°51'41.26"W) (Pérez-Miles 1988, 2 immatures, FCE Ar-1185, misidentified as *Phoneutria* sp.), Meseta de Artigas (31°38'49.78"S; 57°59'48.41"W, 1♂, FCE Ar-7255) or in Esteros de Farrapos National Park (32°40'20.42"S; 58°8'14.67"W, 1♀, FCE Ar-4817). This species is the southernmost representative of the genus which expands its distribution range to the south by the lower course of Paraná and Uruguay rivers (Höfer and Brescovit 2000), through the biological corridor constituted by the riparian forests.

Some recorded species such as *Deinopis amica, Architis capricorna* and *Xiombarg plaumanni* represent the southernmost record for the species. This indicates a limit in the species distribution and a transition between biogeographical regions.

These scenarios occur in others islands of the Uruguay River, for example the record of immature individuals of *Phoneutria* sp. in Pérez-Miles (1988) (probably *Phoneutria nigriventer* (Keyserling 1891)), being the southernmost record in a natural environment for the species. It was also recorded in southern urban localities, such as Montevideo and Buenos Aires, but only by accidental transport in international banana cargo (Simó and Brescovit 2001).

The existence of biological corridors for the spider fauna has already been indicated in the region. Simó et al. (2015) proposed that the truncated hills from northern Uruguay are related with the Aracucaria Forest from southern Brazil, based on the presence of some species in common. This suggestion is supported by geological evidence which indicates an environmental continuity in the past (Perea et al. 2008).

Grela (2004) analysed the floristic geography of tree species of Uruguay and proposed the delimitation of two different dendroflora regions: Western and Oriental. This author recognised in the Western dendroflora a mixed composition with the presence of arboreal species from Paraná and Chaco provinces, being Paraná species that occupy the margins of the Uruguay River and its tributaries. Therefore, the continuous species distribution of the riparian forest is due to the contribution of tropical species from Paraná, which reach the forests of the islands and riverbanks along the Uruguay River. Subsequently, Gutiérrez et al. (2015) identified and delimited the main conservation corridors of Uruguay, establishing them as national connectors to the so-called Uruguay River Valley, based on ecological links at the regional level and trees and birds distribution (Nores et al. 2005, Sganga et al. 1984). To this evidence should be added others, such as those reported by Simó et al. (2014) for harvestmen, in particular the species *Discocyrtus prospicuus* Holmberg, 1876 that is distributed along the riparian forest corridor along the river, according with a Paranaense influence. The authors conclude that the distribution of the opiliofauna is coincident with the distribution of dendroflora proposed by Grela (2004) with the convergence of Pampean and Paranaense biotas.

Therefore, there is an important set of evidence that indicates that the Uruguay River and its associated environments constitute a corridor of fauna and flora, where components of the subtropical biota extend their distribution ranges towards more southern latitudes and temperate climates.

The riparian insular and continental forests, associated with the Uruguay River are considered a priority for conservation (Brazeiro et al. 2015); however, only the implementation of protected areas is not enough to mitigate the loss of biodiversity. It is necessary to change the conservation approach to a larger scale in order to preserve the connection between the areas chosen to be protected (Beier and Noss 1998, Bennett 1999) and even more when the insular and continental riparian forests of Uruguay River along its course, are situated in areas of international limits. Today, only part of the Uruguayan islands is included in protected areas: National Park Esteros de Farrapos and the Uruguay River islands (DINAMA 2014), but others from the upper course of the river remain without official protection. Therefore, as an input for the management and conservation of these areas, it is essential to know the biodiversity they harbour, as well as their dynamics and the flow of biota that exists between them. Our study provides information on the diversity of insular spider fauna from the Uruguay River but other questions need to be answered: How does the taxonomic composition change throughout the river? How is the araneofauna flow across the river? How do periodic floods of the river influence the spider community? Future studies will be necessary to enlarge the knowledge and conservation of the biological linkages in this large river ecosystem.

## Figures and Tables

**Table 1.** Taxonomic list and abundance of the spiders collected in Abrigo island. M: males, F: females, RA: relative abundance, new species records are indicated with an asterisk (*).

	**M**	**F**	**Total**	**RA**
** Anyphaenidae **				
*Otoniela quadrivittata* (Simon, 1897)*		2	2	0.12
*Sanogasta backhauseni* (Simon, 1895)	1		1	0.06
*Sanogasta maculatipes* (Keyserling, 1878)	3		3	0.19
*Tasata parcepunctata* Simon, 1903		2	2	0.12
*Tasata variolosa* Mello-Leitão, 1943	12	4	16	0.99
*Xiruana gracilipes* (Keyserling, 1891)	3	1	4	0.25
*Aysha* sp.1	20	57	77	4.75
*Aysha* sp.2		1	1	0.06
*Aysha* sp.3	1		1	0.06
**Subtotal**	40	67	107	6.60
** Araneidae **				
*Araneus lathyrinus* (Holmberg, 1875)	3	3	6	0.37
*Araneus omnicolor* (Keyserling, 1893)	4	26	30	1.85
*Araneus uniformis* (Keyserling, 1879)	2	5	7	0.43
*Araneus workmani* (Keyserling, 1884)		13	13	0.80
*Cyclosa machadinho* Levi, 1999		6	6	0.37
*Eustala photographica* Mello-Leitão, 1944	20	49	69	4.26
*Eustala taquara* (Keyserling, 1892)		1	1	0.06
*Larinia t-notata* (Tullgren, 1905)	1	13	14	0.86
*Mangora lactea* Mello-Leitão, 1944		15	15	0.93
*Micrathena furcata* (Hahn, 1822)		1	1	0.06
*Nephila clavipes* (Linnaeus, 1767)	32	22	54	3.33
*Ocrepeira venustula* (Keyserling, 1879)	1		1	0.06
*Parawixia audax* (Blackwall, 1863)	5	16	21	1.30
*Parawixia velutina* (Taczanowski, 1878)		1	1	0.06
*Araneus* sp.	3		3	0.19
Araneidae gen. sp.	1	1	2	0.12
**Subtotal**	72	172	244	15.05
** Corinnidae **				
*Creugas lisei* Bonaldo, 2000		2	2	0.12
*Castianeira* sp.1	6	21	27	1.67
*Castianeira* sp.2		1	1	0.06
*Castianeira* sp.3	2		2	0.12
**Subtotal**	8	24	32	1.98
** Ctenidae **				
*Asthenoctenus borelli* Simon, 1897	7	6	13	0.80
**Subtotal**	7	6	13	0.80
** Deinopidae **				
*Deinopis amica* Schiapelli & Gerschman, 1957	2	7	9	0.56
**Subtotal**	2	7	9	0.56
** Desidae **				
*Metaltella simoni* (Keyserling, 1878)	1	2	3	0.19
**Subtotal**	1	2	3	0.19
** Dictynidae **				
*Dictyna* sp.	8	2	10	0.62
**Subtotal**	8	2	10	0.62
** Eutichuridae **				
*Cheiracanthium inclusum* (Hentz, 1847)	4	4	8	0.49
**Subtotal**	4	4	8	0.49
** Gnaphosidae **				
*Apopyllus silvestrii* (Simon, 1905)		1	1	0.06
Gnaphosidae gen. sp.		1	1	0.06
**Subtotal**	0	2	2	0.12
** Hahniidae **				
Hahniidae gen. sp1	34	31	65	4.01
Hahniidae gen .sp2	25	31	56	3.46
Hahniidae gen. sp.3		1	1	0.06
**Subtotal**	59	63	122	7.53
** Linyphiidae **				
*Dubiaranea difficilis* (Mello-Leitão, 1944)*	19	43	62	3.83
*Scolecura parilis* Millidge, 1991*	19	13	32	1.98
*Sphecozone venialis* (Keyserling, 1886)*	6	15	21	1.30
*Erigone* sp.	3		3	0.19
*Psilocymbium* sp.	2	3	5	0.31
*Scolecura* sp.	22	18	40	2.47
*Sphecozone* sp.	1		1	0.06
*Tutaibo* sp.		1	1	0.06
Linyphiidae gen. sp.1	3	5	8	0.49
Linyphiidae gen. sp.2	9	7	16	0.99
Linyphiidae gen. sp.3	27	9	36	2.22
Linyphiidae gen. sp.4	7	15	22	1.36
Linyphiidae gen. sp.5	24	2	26	1.60
Linyphiidae gen. sp.6	41	5	46	2.84
Linyphiidae gen. sp.7	3	2	5	0.31
Linyphiidae gen. sp.8		3	3	0.19
Linyphiidae gen. sp.9	2		2	0.12
Linyphiidae gen. sp.10	6	3	9	0.56
Linyphiidae gen. sp.11	1		1	0.06
**Subtotal**	195	144	339	20.93
** Lycosidae **				
*Agalenocosa pirity* Piacentini, 2014*	2	2	4	0.25
*Agalenocosa velox* (Keyserling, 1891)	1		1	0.06
*Lobizon corondaensis* (Mello-Leitão, 1941)*		1	1	0.06
*Lobizon humilis* (Mello-Leitão, 1944)	57	9	66	4.07
*Lycosa poliostoma* (C. L. Koch, 1847)	1		1	0.6
*Lycosa thorelli* (Keyserling, 1877)	5	22	27	1.67
*Lycosa aff. thorelli*	16		16	0.99
*Allocosa* sp.		1	1	0.06
**Subtotal**	82	35	117	7.22
** Mimetidae **				
*Mimetus melanoleucus* Mello-Leitão, 1929*	1	1	2	0.12
**Subtotal**	1	1	2	0.12
** Mysmenidae **				
*Microdipoena* sp.	4	4	8	0.49
**Subtotal**	4	4	8	0.49
** Oonopidae **				
*Neotrops lorenae* Grismado & Ramírez, 2013	1		1	0.06
*Neotrops sciosciae* Grismado & Ramírez, 2013		1	1	0.06
*Xiombarg plaumanni* Brignoli, 1979*		3	3	0.19
*Gamasomorpha* sp.	6	6	12	0.74
*Neoxyphinus* sp.	29	8	37	2.28
**Subtotal**	36	18	54	3.33
** Pholcidae **				
*Mesabolivar uruguayensis* Machado, Laborda, Simó & Brescovit, 2013	12	36	48	2.96
**Subtotal**	12	36	48	2.96
** Pisauridae **				
*Architis capricorna* Carico, 1981*	6	8	14	0.86
**Subtotal**	6	8	14	0.86
** Salticidae **				
*Aphirape flexa* Galiano, 1981	1	3	4	0.25
*Cotinusa trifasciata* (Mello-Leitão, 1943)*	2		2	0.12
*Dendryphantes mordax* (C. L. Koch, 1846)	3		3	0.19
*Hisukattus transversalis* Galiano, 1987	29	25	54	3.33
*Lyssomanes pauper* Mello-Leitão, 1945	1	2	3	0.19
*Synemosyna aurantiaca* (Mello-Leitão, 1917)*	1		1	0.06
*Ashtabula* sp.	5		5	0.31
*Bellota* sp.		1	1	0.06
*Cotinusa* sp.1	2		2	0.12
*Cotinusa* sp.2		1	1	0.06
*Pensacola* sp.	2		2	0.12
Salticidae gen. sp.1		2	2	0.12
Salticidae gen. sp.2	1		1	0.06
Salticidae gen. sp.3		1	1	0.06
Salticidae gen. sp.4		3	3	0.19
Salticidae gen. sp.5		1	1	0.06
**Subtotal**	47	39	86	5.31
** Segestriidae **				
*Ariadna mollis* (Holmberg, 1876)	1	2	3	0.19
**Subtotal**	1	2	3	0.19
** Sparassidae **				
*Polybetes pythagoricus* (Holmberg, 1875)	1		1	0.06
**Subtotal**	1	0	1	0.06
** Tetragnathidae **				
*Glenognatha lacteovittata* (Mello-Leitão, 1944)	5	2	7	0.43
*Leucauge volupis* (Keyserling, 1893)*	6	50	56	3.46
Tetragnathidae gen. sp.		1	1	0.06
**Subtotal**	11	53	64	3.95
** Theridiidae **				
*Anelosimus vierae* Agnarsson, 2012	1		1	0.06
*Cryptachaea altiventer* (Keyserling, 1884)*		8	8	0.49
*Cryptachaea bellula* (Keyserling, 1891)*	2	3	5	0.31
*Theridion cf. positivum* Chamberlin, 1924	4	2	6	0.37
*Thymoites piratini* Rodrigues & Brescovit, 2015		3	3	0.19
*Thymoites puer* (Mello-Leitão, 1941)	5	4	9	0.56
*Argyrodes* sp.	8	7	15	0.93
*Cryptachaea* sp.	5	9	14	0.86
*Euryopis* sp.	4	3	7	0.43
*Guaraniella* sp.1	2	3	5	0.31
*Guaraniella* sp.2	1	6	7	0.43
*Theridion* sp.1	1		1	0.06
*Theridion* sp.2		2	2	0.12
*Thymoites* sp.1	1	3	4	0.25
*Thymoites* sp.2		7	7	0.43
Theridiidae gen. sp.1	26	57	83	5.12
Theridiidae gen. sp.2		4	4	0.25
Theridiidae gen. sp.3		2	2	0.12
Theridiidae gen. sp.4		1	1	0.06
Theridiidae gen. sp.5		12	12	0.74
Theridiidae gen. sp.6	2		2	0.12
Theridiidae gen. sp.7		5	5	0.31
Theridiidae gen. sp.8	1	2	3	0.19
Theridiidae gen. sp.9		4	4	0.25
Theridiidae gen. sp.10	4		4	0.25
Theridiidae gen. sp.11	5	29	34	2.10
Theridiidae gen. sp.12		2	2	0.12
Theridiidae gen. sp.13		1	1	0.06
Theridiidae gen. sp.14	1		1	0.06
Theridiidae gen. sp.15		1	1	0.06
Theridiidae gen. sp.16		1	1	0.06
Theridiidae gen. sp.17		1	1	0.06
Theridiidae gen. sp.18	2		2	0.12
**Subtotal**	75	182	257	15.86
** Thomisidae **				
*Misumenops maculissparsus* (Keyserling, 1891)		1	1	0.06
Sidymella cf. lucida (Keyserling, 1880)	1		1	0.06
Titidius aff. albipes	6	4	10	0.62
Tmarus aff. stiliferus	1		1	0.06
*Metadiaea* sp.		3	3	0.19
*Misumenoides* sp.	1		1	0.06
*Synaema* sp.	2		2	0.12
*Tmarus* sp.1	2		2	0.12
*Tmarus* sp.2		4	4	0.25
Thomisidae gen. sp.		1	1	0.06
**Subtotal**	13	13	26	1.60
** Trachelidae **				
*Meriola cetiformis* (Strand, 1908)	5	4	9	0.56
*Trachelopachys keyserlingi* (Roewer, 1951)	1		1	0.06
*Trachelopachys* sp.		1	1	0.06
**Subtotal**	6	5	11	0.68
** Trechaleidae **				
*Paratrechalea ornata* (Mello-Leitão, 1943)	19	19	38	2.35
**Subtotal**	19	19	38	2.35
** Uloboridae **				
*Uloborus elongatus* Opell, 1982*	1	1	2	0.12
**Subtotal**	1	1	2	0.12
**Grand total**	711	909	1620	100

**Table 2.** Abundance (Ab.), relative abundance (RA), species richness (Sp. rich.) and relative species richness (RR) per guild of the spiders collected in Abrigo island.

**Guilds**	**Ab.**	**RA**	**Sp. rich.**	**RR**
Ground hunters (GH)	959	12.6	22	15.2
Ambush hunters (AH)	541	7.1	11	7.6
Sensing web weavers (SEW)	5	0.1	1	0.7
Space web weavers (SPW)	1977	26.0	37	25.5
Orb web weavers (OW)	1682	22.1	20	13.8
Sheet web weavers (SHW)	802	10.5	24	16.6
Specialists (S)	165	2.2	2	1.4
Other hunters (OH)	1474	19.4	28	19.3

## Hosting institution

Sección Entomología, Facultad de Ciencias, Universidad de la República. Iguá 4225. CP 11400. Montevideo. Uruguay.
